# Lipid-based nanoparticles via nose-to-brain delivery: a mini review

**DOI:** 10.3389/fcell.2023.1214450

**Published:** 2023-08-22

**Authors:** Yang Xinchen, Tan Jing, Guan Jiaoqiong

**Affiliations:** ^1^ Department of Neurology, West China Hospital, Sichuan University, Chengdu, China; ^2^ Department of Rehabilitation Medicine, Guangzhou First People’s Hospital, School of Medicine, South China University of Technology, Guangzhou, China

**Keywords:** nanoparticles, nose-to-brain, blood-brain barrier, drug delevery, central nervous system

## Abstract

Central nervous system disorders significantly affect the lives and health of millions of people worldwide. Despite many therapeutic drugs are available that could potentially target central nervous system disorders, their clinical utility is severely constrained by their inability to cross the blood-brain barrier (BBB). Fortunately, nanotechnology has been advanced to offers a solution to allow drugs reaching the targeted brain regions safely, efficiently, and precisely through nasal drug delivery system (NDDS), bypassing the BBB completely. This strategy can promote the drug accumulated in the targeted brain region, improve drug bioavailability, and minimal side effects and mucociliary clearance effectively. In this review, we elaborate recent advances in the use of lipid-based nanoparticles, involving liposomes, nanoemulsions, nanostructured lipid carriers, and solid lipid nanoparticles. Besides, we particularly introduced the nasal cavity physiological structure, and further summarized the nose-to-brain drug delivery pathways, including olfactory, trigeminal, and blood circulation pathway. Moreover, the mechanism and route of NDDS by various types of nanoparticles are also highlighted.

## 1 Introduction

Central nervous system disorders are a subset of neurological illnesses affecting the structure or function of the brain and spiral cord. These disorders mainly include neurodegenerative diseases, functional disorders, structural disorders, CNS infections, and demyelinating diseases (MOUJALLED et al., 2021). The most commonly recognized ones are Alzheimer’s disease (AD), Parkinson’s disease (PD), depression, schizophrenia, epilepsy, stroke, as well as primary brain tumors (Glioblastoma, etc.) (GOEDERT and SPILLANTINI, 2006; BUSH et al., 2017; BALESTRINO and SCHAPIRA, 2020). Drug therapies are often used to cure or slow the progression of CNS disorders (e.g., levodopa is used to treat PD, which works by increasing the levels of dopamine (TAMBASCO et al., 2018)). Currently, the route of administered orally are exposed to first-pass hepatic metabolism and require frequent administrations to ensure the constant presence of the drug at the site of action, the amount of drug that reaches the target brain region is constrained by the low diffusion coefficient of the BBB, which decline the therapeutic effect. As a result of the increased prevalence of CNS disorders, there is a growing demand for safer, quicker, and more efficient therapies.

The biggest hindrance in the treatment of the CNS disorders is the BBB, a dynamic and complex neural functional unit that interacts with the glial system (ZHAO et al., 2021). It is not only composed of brain microvascular endothelial cells (BMECs), but also the pericytes, astrocytes and basal membrane that are involved in the maintenance of BBB function. BMECs form a tight capillary endothelium and are the major part of the BBB. The end-feet of astrocytes wrap BMECs, and pericytes are embedded in the basement membrane which is constituted by matrix protein secreted from cells. BBB plays a vital role in maintaining the homeostasis of the CNS and protecting the CNS from toxins, pathogens, inflammation, injury, and disease (DANEMAN and PRAT, 2015). Due to the poor phagocytosis of endothelial cells, the presence of highly expressing ATP-binding cassette transporters and a large number of tightly connected cells, small molecules such as oxygen and carbon dioxide or lipophilic molecules can permeate BBB through free diffusion, while over 98% biomolecular pharmaceuticals and all macromolecular neurotherapy medications cannot enter the brain from the blood due to this strong barrier capacity (PARDRIDGE, 2005; BANKS and ERICKSON, 2010; CROWE et al., 2018). Because of this characteristic, a large number of promising drugs cannot be transported to the CNS and maintain a certain concentration (FAN et al., 2018), or they need to be modified to meet the specific transport mechanism (e.g., limiting the drug molecule to be highly lipophilic and molecular weight <500 Da, receptor-mediated endocytosis, and carrier-mediated transport) (LEE D. and MINKO T., 2021), which creates a lot of inconvenience and challenges. Among various delivery strategies, NDDS is receiving increasing attention because it can bypass the BBB and allow drugs to be transported directly through neuronal pathways, which will be highly beneficial for treating CNS disorders.

When NDDS was first identified (in 1989), an increasing number of research have been conducted in an effort to discover more effective CNS disorders therapies. Compared normal orality delivery, NDDS shows better penetration, more excellent patient compliance, also can bypass the hepatic first-pass effects, and rapid absorption and onset of action (TALEGAONKAR and MISHRA, 2004; SCHWARZ and MERKEL, 2019). Based on this, a variety of novel NDDS have been designed and developed for breaking through delivery barriers and treating the CNS disorders (ARORA D. et al., 2020; DHURI et al., 2021; PANZARINI et al., 2021). Some drugs have been designed to traditional nasal delivery forms include nasal drops, sprays, powders and so on (GHORI et al., 2015). However, due to the mucociliary clearance system in the nasal cavity, there is a risk of drugs being rapidly cleared. Therefore, a simple NDDS administration method is not effective enough to effectively enter the target brain region, and cannot exert its efficacy well. Notably, with the continuous breakthrough of nanomaterial technology, nanomaterial encapsulated drugs combined with NDDS have provided a broader prospect for the treatment of CNS disorders.

Nanomaterials are excellent carriers for drug delivery, and their remarkable advantages are reflected in high specific surface area, excellent modifiability, long circulation and low toxicity (MARIANECCI et al., 2017; HOU et al., 2020; SA et al., 2022). Encapsulating drugs (e.g., small molecule compounds or proteins) in nanoparticles carriers and administering them through the nasal cavity can greatly improve the efficiency of medications entering the CNS. And nose-to-brain targeted nanoparticles delivery has made remarkable progress since its discovery (GAO, 2016). With the further exploration of NDDS by researchers, some new preparation processes and nanocarriers such as liposomes, nanoemulsions, nanostructured lipid carrier and solid lipid nanoparticles having showed potential in keeping poorly lipophilic medicines from penetrating BBB in various fields of clinical treatment of CNS disorders (WEN et al., 2017; BAJRACHARYA et al., 2019; LONG et al., 2020; ANTIMISIARIS et al., 2021; COSTA et al., 2021; PATEL et al., 2021). Another improved role is the sustained or delayed release of the nanocarriers, which enables steady and prolonged drug exposure to nasal mucosa (SONVICO et al., 2018). When nanoparticles are administered intranasally, they are quickly cleared from the nasal cavity due to the mucociliary clearance system (CUNHA et al., 2017). However, if the nanoparticles are formulated to release slowly or if they are coated with the mucoadhesive material, they can adhere to the nasal mucosa and remain in contact with it for a longer period of time (ZAMAN et al., 2018). The nasal mucosa is more likely to absorb nanoparticles as a benefit of this longer contact period, which may enhance drug delivery and therapeutic efficacy. Furthermore, nanocarriers can protect the drugs. For example, peptide drugs are easily degraded by enzymes in the nasal cavity under the traditional formulations, but the new NDDS can protect them from being metabolized by the nasal cavity and improve their bioavailability (AHLAWAT et al., 2020).

In order to better develop and improve the nanoparticles through NDDS, this article summarizes the physiological structure of the nasal cavity, nose-to-brain delivery pathways, lipid-based nanocarriers and their application in the treatment of CNS disorders.

## 2 Physiological structure and absorption characteristics of the nasal cavity

### 2.1 Nasal structure and function

The nose is the main organ of olfaction and respiration (BONFERONI et al., 2019). And the nasal septum separates the human nasal cavity into two channels, which extend from the nostrils all the way towards the nasopharyngeal tract. The main cavity of the turbinate is about 5–8 cm, and its absorption surface area is about 150–200 cm^2^ (COSTA et al., 2021). According to its function and organization, the nasal cavity can be divided into vestibular area, respiratory area and olfactory area. The vestibular region is a slight expansion inside the nostrils and before the main nasal cavity, which has a small surface area and little absorptive function (BOURGANIS et al., 2018). The respiratory area contains a large number of epithelial cells, including pharyngeal cells, cilia cells, intermediate cells and basal cells (ANTIMISIARIS et al., 2021). The mucosal surface cells are covered with many microhairs and abundant nasal glands, which effectively increase the drug absorption effect. Therefore, drugs are mainly absorbed in the respiratory area and enter the systemic circulation (SONVICO et al., 2018; PATEL et al., 2021). Associated with CNS administration is mainly the olfactory region, which is composed of sustentacular cells, basal cells, and olfactory sensory neurons (OSNs). Among them, OSNs are specialized bipolar neurons located between supporting cells and are composed of three parts: the cell body, dendrites and axons (MAIGLER et al., 2021). After extending through the basal layer of the epithelium, the central processes of olfactory cells converge to form olfactory filament nerve bundles (HARKEMA et al., 2006). The olfactory filament is surrounded by olfactory ensheathing cells (OEC) and olfactory nerve fibroblasts to form the nerve sheath (FIELD et al., 2003). The olfactory filament passes through the cribriform plate of the ethmoid bone into the brain and terminates in the olfactory bulb (FENG et al., 2018). Therefore, this region is directly related to the nasal administration into the brain.

### 2.2 Nose-to-brain delivery pathways

The physiological anatomy of the nasal cavity and brain provides physiological possibilities for NDDS. Following nasal administration, drug molecules are transported to the entrance near the pial brain surface in the cranial compartment after they cross the epithelial barrier (olfactory or respiratory) in the nasal passages in different ways (MAIGLER et al., 2021). Then they are transported from these initial brain entry sites into other tissues within the CNS.

**FIGURE 1:**
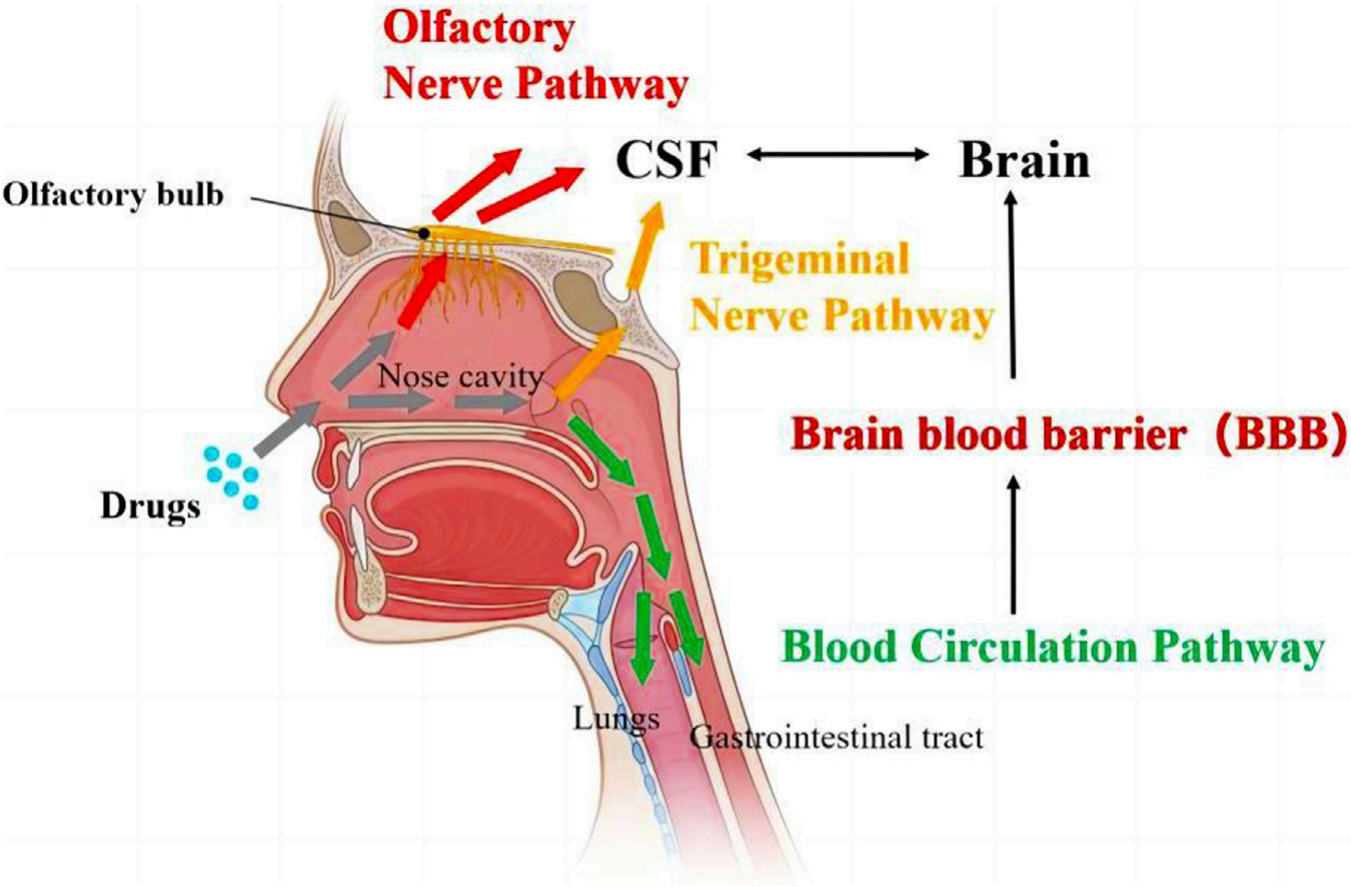
Transport pathways of drugs from nose-to-brain (ALBERTO et al., 2022).

At present, the transport pathways (Figure 1) are divided into the following three types: olfactory nerve pathway, trigeminal nerve pathway, and blood circulation pathway (COSTA et al., 2019). After being absorbed into the nasal region, the drugs can pass through the olfactory bulb or the trigeminal nerve to reach the cerebrospinal fluid, where they are capable of further entering the brain for therapeutic purposes. Alternatively, it may enter the systemic circulation via the lungs or gastrointestinal tract, ultimately overcoming the BBB to gain access to the brain.

#### 2.2.1 Olfactory pathway

**FIGURE 2:**
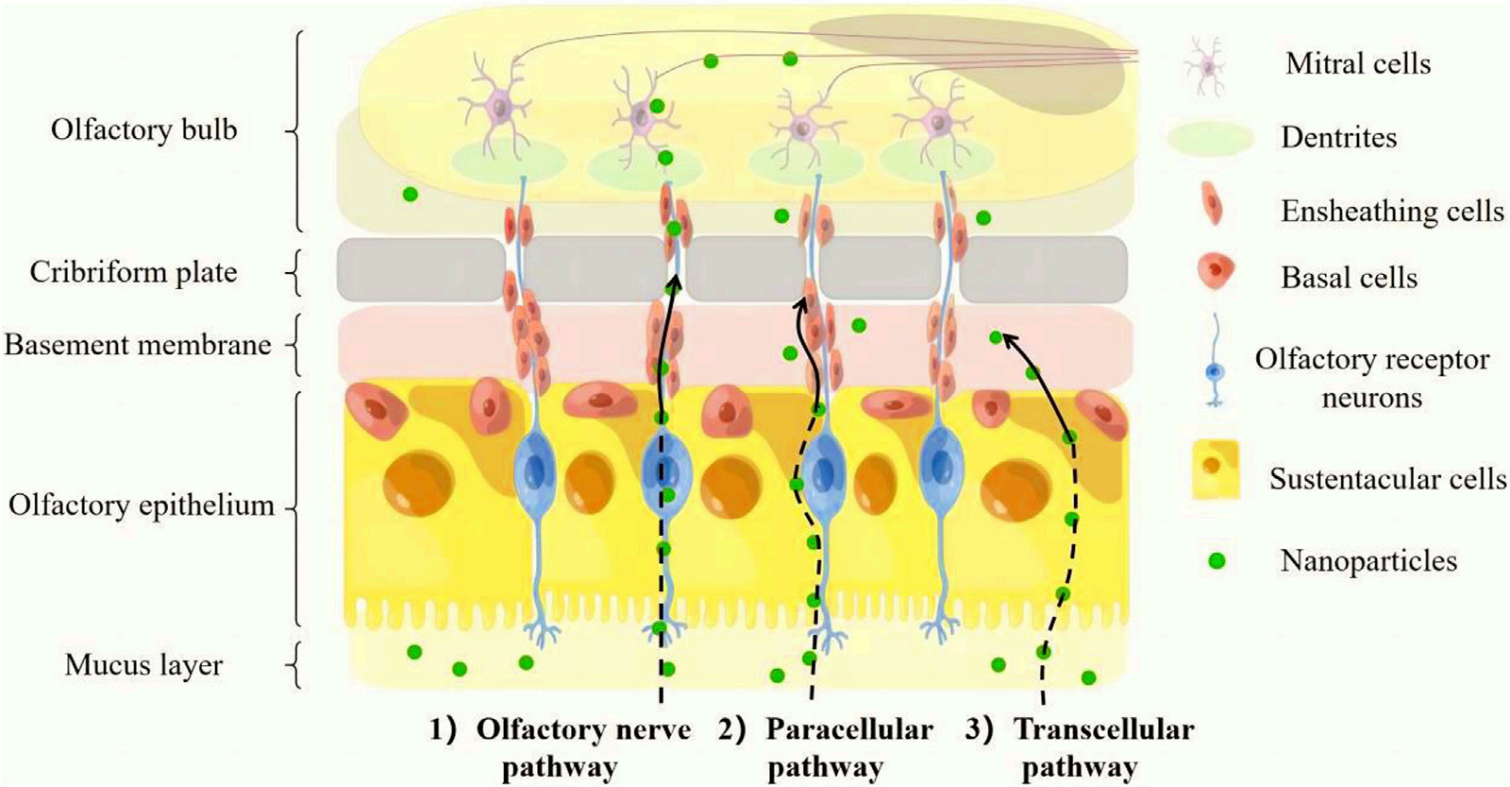
Mechanisms involved in direct nose-to-brain nanoparticles transport from the olfactory region.

The olfactory system consists of two parts: the intracellular pathway and the extracellular pathway (PARDESHI and BELGAMWAR, 2013) (Figure 2). The intracellular pathway, also known as the olfactory nerve pathway, starts when an olfactory receptor neuron internalizes a nanoparticle, after which an endocytic vesicle traffics within the neuron’s cells such as OEC, and ultimately the molecule is released through exocytosis by mitral cells (SINGH et al., 2020). The extracellular pathway contains two parts, respectively paracellular pathway and transcellular pathway. The olfactory epithelium can be crossed through a paracellular pathway, which involves passing through a gap in the sustentacular cells (SUS) and basement membrane. Unlike the transcellular pathway, which relies on receptor-mediated endocytosis or passive diffusion, the paracellular pathway does not require receptor binding and it is best suited for small, hydrophilic molecules. On the other hand, the transcellular pathway is suitable for hydrophobic nanoparticles and occurs through receptor-mediated endocytosis or passive diffusion of nanoparticles across the SUS membrane (LEE D. and MINKO T., 2021).

#### 2.2.2 Trigeminal pathway

The trigeminal nerve is the fifth cranial nerve and is the thickest nerve on the face, giving three large branches composed of peripheral processes from the trigeminal node anteriorly, followed by the ophthalmic nerve, maxillary nerve, and mandibular nerve inside-out (TERRIER et al., 2022). Among them, ophthalmic nerve branches and maxillary nerve branches extend into the epithelial cells of the olfactory and respiratory regions of the nasal cavity, and the other end enters the CNS at the pontine site and terminates in the spinal nucleus of the trigeminal nerve in the brain cadre (SCHAEFER et al., 2002). The substance can be carried into the brain by the intra-axonal pathway of neurons once it has reached the trigeminal branch of the olfactory and respiratory regions (TUCKER, 1971; ANTON and PEPPEL, 1991; THORNE et al., 2004). Although being a less significant drug delivery mechanism than the olfactory pathway, the trigeminal pathway also offers a new perspective on how to introduce drug directly entry into the brain (COSTA et al., 2019).

#### 2.2.3 Blood circulation pathway

The blood circulation pathway is a complex network of blood vessels and organs that work together to transport blood, oxygen, and nutrients throughout the body. Because there are abundant blood vessels and lymphatic vessels distributed in the lamina propria of the nasal mucosa, in which blood flow is abundant, the substance can be transported into the circulatory system and later reach the brain through the BBB for low molecular weight lipophilic molecules and medications (BOURGANIS et al., 2018). And the blood circulation pathway by NDDS has several advantages over intravenous injection, including faster absorption, non-invasive delivery, avoidance of first-pass metabolism, and reduced systemic exposure.

Although the nasal route of administration is a non-invasive mode of administration that can overcome BBB effectively, only a limited number of drugs can be absorbed directly into the brain using this route. For drugs which have high hydrophilic or molecular weight that make them difficult to penetrate BBB. Scientists have combined nanotechnology with nasal drug delivery to solve this problem. There are different types of common nanocarriers such as polymers, lipids and inorganic nanoparticles. Nanocarriers can encapsulate drugs and protect them from degradation and clearance by the body’s immune system, thus prolonging their circulation time and increasing their chances of crossing the BBB. In addition, another advantage is to use surface modifications to enhance the ability of nanoparticles to cross the BBB. This can be achieved by coating the nanoparticles with specific ligands or antibodies that can interact with transporters or receptors expressed on the endothelial cells of the BBB, facilitating their transcytosis across the barrier.

## 3 Lipid-based nanocarriers and its applications via NDDS for CNS disorders treatment

Considering the safety and neurotoxicity of nanoparticles during drug delivery, lipid-based nanocarriers are of great advantage in drug delivery for central system diseases due to their low toxicity and stability. Researchers have developed lipid-based nanocarriers using physiological lipids such as liposomes, nanoemulsions, nanostructured lipid carrier and solid lipid nanoparticles (Supplementary Figure S1).

### 3.1 Liposomes

Liposomes are lipid-based vesicles in the nanometric range (EMAD et al., 2021), and the drug is delivered into the interior of the cell through the fusion of liposomes and the cell membrane. Encapsulating the drug into liposomes can reduce the direct contact between the drug and nasal mucosa while the lipid bilayer can regulate and control the release and uptake of the drug to achieve the effect of sustained release. At the same time, they can encapsulate lipophilic and hydrophilic molecules and have a wide range of uses. Several available pre-clinical studies have shown that liposomal formulations delivered nasally can increase the efficiency of drug transport into the brain, thereby improving drug utilization and reducing drug-related systemic side effects, thereby improving drug efficacy (VIEIRA and GAMARRA, 2016; BOURGANIS et al., 2018; ERDO et al., 2018).

Saka et al. (SAKA et al., 2021) prepared liposomes of imatinib mesylate for Alzheimer’s disease (AD) with particle size less than 150 nm and sustained drug release up to 96 h. The liposomal drug formulation was compared with the normal drug solution to determine its cytotoxicity to mouse neuroblastoma N2a cells and showed no toxicity at concentrations up to 25 μg/mL. The brain deposition is evaluated in comparison with these two kinds of formulation in rats. The experiments showed that liposomes effectively improved brain deposition compared to common drug solution by area under curve (AUC). These results suggest that nose-to-brain administration of imatinib mesylate liposomes improved drug permeation and residence time in the brain compared to oral administration.

Katona et al. (KATONA et al., 2022)developed liposomes of lomustine (LOM) and *n*-propyl gallate (PG) for targeting glioblastoma multiforme (GBM) by nose-to-brain delivery. The combination of PG and LOM in liposomes and nose-to-brain delivery can overcome the problems of poor water solubility, poor absorption properties, and toxicity in the circulation. Liposomal carriers were then optimized and characterized, the experiments showed suitable particle size (127 ± 6.9 nm), zeta potential (−34 ± 1.7 mV), and high encapsulation efficacy (63.57% ± 1.3% of PG and 73.45% ± 2.2% of LOM) meeting the acceptance criteria of nose-to-brain transport for both drugs. MTT assays of the PG-LOM formulation were also performed *in vivo*, demonstrating reduced antiproliferative effects on all cell types.

These research works provide valuable information for the administration of liposomes in NDDS. Liposomes, which improve drug permeability and have the potential to be surface functionalized with different ligands, are gaining attention as promising strategies for the treatment of neurological diseases (PASHIROVA et al., 2018).

### 3.2 Nanoemulsions

Nanoemulsions (NEs) are nano-scale colloidal systems containing an oil phase, an aqueous phase, and a surfactant, usually with particle sizes in the range of 20–200 nm. There are three types of nanoemulsions: water-in-oil, oil-in-water, and bicontinuous nanoemulsions. Benefit from their superior ability to increase drug solubility, provide a large surface area for drug absorption and easy preparation (LEE DV. and MINKO T., 2021), NEs have received a lot of attention. Previous findings have shown that excipients contained in NEs can facilitate their penetration through the nasal epithelium to the brain. For example, chitosan can prolong the residence time of NEs in the nasal cavity and promote drug delivery from the nose to the brain delivery (AHMAD et al., 2017). However, NEs are not a thermodynamically stable system and may break up during delivery, resulting in the efflux of internally encapsulated moieties.

Tetrabenazine is an effective treatment for Huntington’s disease, but it has low oral bioavailability. Arora (ARORA A. et al., 2020) prepared tetrabenazine loaded nanoemulsion for intranasal administration and optimized the formulation of nanoemulsion using the quality by design technique. After optimization, the particle size was 106.80 ± 1.96 nm, the polydispersity index value was 0.198 ± 0.005 and a zeta potential was −9.63 ± 0.63 mV. The formulation was found to have a 1.68-fold increase in permeation rate compared to the tetrabenazine suspension. MTT assays performed on neuro2a cell lines showed that nanoemulsion loaded with tetrabenazine showed better cell viability than placebo and aqueous solutions.

### 3.3 Solid lipid nanoparticles

Solid lipid nanoparticles (SLNs) are a new generation of lipid-based nanocarriers, which are nanoemulsions formed by replacing liquid lipids with solid lipids. They usually have a diameter of 100–300 nm and are structured with a hydrophobic core inside and a single lipid coat outside (LEE DV. and MINKO T., 2021), with some hydrophobic chains of lipids embedded in the solid to allow better drug dispersion (PRAGATI et al., 2009). The basic preparation methods include high-pressure homogenization, ultrasonication, microemulsion, double emulsion, and emulsification solvent evaporation (PRAGATI et al., 2009; SINGH et al., 2010). SLNs have several advantages in drug delivery: they can achieve slow and controlled release for drug delivery, have high physicochemical stability and low biotoxicity (ANNUREHMAN et al., 2019). The main disadvantages of SLNs are their limited choice of shapes and non-uniform particle formation due to colloidal polymerization, which may further lead to explosive drug release (PURI et al., 2009; GHASEMIYEH and MOHAMMADI-SAMANI, 2018).

Rotigotine (RTG), used for the treatment of Parkinson’s disease, has a low oral bioavailability and is limited in its clinical use. Patel (PATEL et al., 2020) prepared solid lipid nanoparticles (RTG-SLNs) of RTG using a hot melt emulsification method in which Dynasan 118 and Tween 80 were selected and optimized as solid lipid and surfactant, respectively. The optimized RTG-SLNs had a particle size (129 nm), polydispersity index (0.285), zeta potential (−23.1 mV) and *in vitro* drug release (99%) in phosphate buffer 7.4 for 30 h.

### 3.4 Nanostructured lipid carriers

Nanostructured lipid carriers (NLCs) are lipid nanoparticles that have been developed in recent years to overcome the drawbacks of SLNs (LEE DV. and MINKO T., 2021). It consists of a combination of solid and liquid lipids (NGUYEN et al., 2022), and since hydrophobic molecules are more soluble in liquid lipids than in solid lipids, NLCs can achieve higher encapsulation efficiency compared to SLNs (AGRAWAL et al., 2020). NLCs are made less toxic and stable by using physiological lipids such as mono-, di-, and triglycerides, fatty acids, and waxes (ALAM et al., 2014). Overall, their advantages in terms of controlled drug release, increased stability, high loading, and low toxicity make them promising targeting vehicles for the treatment of various diseases (NASIRIZADEH and MALAEKEH-NIKOUEI, 2020; RAHMAN et al., 2020). However, NLCs have certain limitations, including low encapsulation efficiency when two or more therapeutic drugs are used in combination and poor loading capacity of hydrophilic drugs (TAPEINOS et al., 2017).

Noorulla (NOORULLA et al., 2022) prepared chitosan (CH)-decorated nanostructured lipid carriers (BPE-CH-NLCs) loaded with buspirone for nasal brain delivery. The authors used solvent diffusion evaporation method to prepare nanocarriers coated with CH and continuously evaluated to optimize the formulation. Among them, a mixture of glycerol monostearate (GMS) and oleic acid was used as lipid and Tween 80 was used as surfactant. The experimental results showed that the optimized NLCs had particle size (190.98 ± 4.72 nm), zeta potential (+17.47 mV) and encapsulation efficacy (80.53% ± 1.26% w/w) with an AUC value intranasal of 3.06-fold for intravenous administration of BPE-CH-NLCs, and 2.17-fold compared to intranasal administration of BPE-Sol. This study suggests that GMS-oleic acid-based NLCs coated with CH may be an effective nanocarrier for nasal administration of BPE to the brain.

In addition, given the greater prevalence of CNS disorders, there is a growing need for safer, quicker, and more efficient treatments. When the benefits of NDDS are considered alongside the features of nanoparticles, the creation of novel formulas containing nanoparticles for NDDS appears to have great promise as an option to current therapies. The applications of nanocarrier medicines for the therapy of CNS disorders via NDDS are summarized in Table 1.

**TABLE 1:** Applications of nanoparticle in nose-to-brain delivery for therapeutic CNS disorders.

Drug	Nanocarrier	Targeted disease	Major results	References
Imatinib mesylate	Liposome	Alzheimer’s disease	These liposomal formulations have a controlled release time of 96 h, and the results indicated that nose-to-brain application of CDPC liposomes increased the brain bioavailability of IMB by seven folds when compared to oral and intranasal solution groups	ERDO et al. (2018)
Lomustine and n-propyl gallate	Liposome	Glioblastoma multiforme	The combination of PG and LOM in liposomes and nose-to-brain delivery can surmount the issues of low water solubility, weak absorption characteristics and circulation toxicity. MTT assays of the PG-LOM formulation were also performed *in vivo*, demonstrating reduced antiproliferative effects on all cell types	VIEIRA and GAMARRA (2016)
Tetrabenazine	Nanoemulsions	Huntington’s disease	Comparison to the tetrabenazine solution, the formulation was found to have a 1.68-fold increase in permeation rate. And tetrabenazine-loaded nanoemulsion outperformed control and aqueous solutions in MTT assays performed on neuro2a cell lines in terms of cell viability	LEE and MINKO (2021b)
Risperidone	Solid Lipid Nanoparticles	Schizophrenia	RSLNs showed higher hindlimb retraction time indexes compared to RSP solution through mouse foot and paw test, indicating higher brain targeting of RSLNs. The concentration of risperidone SLN delivered via the IN group was significantly higher than that of the risperidone IV group and slightly higher than that of the risperidone SLNs IV group. The concentration of risperidone in the blood of SLN IN is approximately is one-half that of the IV SLN, which implies that less risperidone can enter the body circulation and reduce its systemic side effects	PURI et al. (2009)
Buspirone	Nanostructured Lipid Carriers	Anxiety disorder	The BPE-CH-NLCs intranasally with an AUC value of 3.06-fold for intravenous administration of BPE-CH-NLCs, and 2.17-fold compared to intranasal administration of BPE-Sol	RAHMAN et al. (2020)

## 4 Conclusion

Developing novel and efficient drug delivery administration is essential to handle the current clinical therapy of CNS disorders. NDDS can avoid liver metabolism and bypass BBB obstruction, allowing drug molecules to enter the brain through olfactory never pathway, trigeminal never pathway, and blood circulation pathway, and acting on target neurons. However, this approach works best when the drug is used in a higher concentrations. Fortunately, lipid-based nanocarrier system can greatly enhance the drug concentration in the nasal cavity. The drug is wrapped into liposomes, NEs, SLNs, NLCs, etc. To protect the drug from being degraded by nasal mucosa and achieve the drug’s brain targeting. The disadvantage lies in the high cost of nanomaterials and the temporary inability to generate them in large quantities.

In conclusion, the combination of nose to brain delivery systems and nanocarrier systems is a very promising drug delivery method for treating CNS disorders. It can minimize drug dosage, toxic and side effects, and maximize bioavailability by preventing drugs from being blocked by the BBB. Further research is needed to customized drug administration system based on drug characteristics, nasal characteristics, and nanocarrier characteristics can be developed and refined to offer trustworthy new therapy approaches for CNS disorders.
