# Supplementary figures and images for: Lipid-based nanoparticles via nose-to-brain delivery: a mini review
Source: Front Cell Dev Biol. 2023 Aug 22;11:1214450. doi: 10.3389/fcell.2023.1214450 (PMC10477605; doi:10.3389/fcell.2023.1214450)

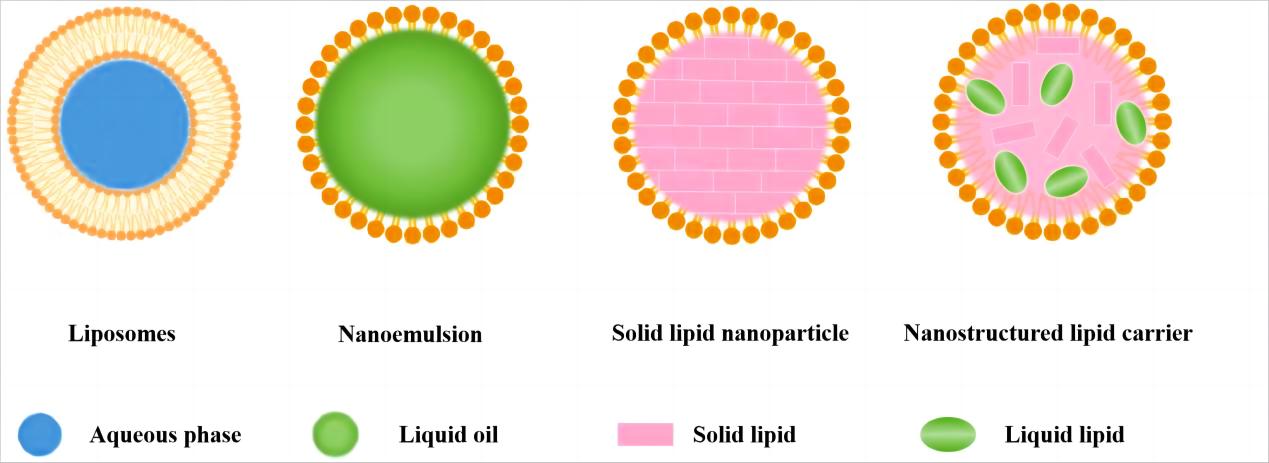

Supplement: Supplementary file 1 [file Image1.JPEG]
